# STING activation increases the efficiency of temozolomide in PTEN harbouring glioblastoma cells

**DOI:** 10.55730/1300-0144.5828

**Published:** 2024-01-21

**Authors:** Zafer YILDIRIM, Eda DOĞAN, Hale GÜLER KARA, Buket KOSOVA, Vildan BOZOK

**Affiliations:** 1 Department of Medical Biology, Faculty of Medicine, Ege University, İzmir, Turkiye; 2 Department of Medical Biology, Faculty of Medicine, Harran University, Şanlıurfa, Turkiye

**Keywords:** cGAS/STING pathway, 2′3′-c-di-AM(PS)2 (Rp,Rp), STING agonist, PTEN, glioblastoma, temozolomide

## Abstract

**Background/aim:**

Glioblastoma is one of the most aggressive tumours, resistant to all applied therapy regiments and prone to relapse. Median survival rates are therefore only expressed as months. STING agonists are immunomodulatory molecules that activate type I interferon expression, making them potentially useful in regulating the tumour microenvironment. Since PTEN serves as a critical phosphatase in activating interferon-regulating transcription factors and is frequently mutated in glioblastoma cells, this study aimed to investigate STING activation in glioblastoma cell lines, examining whether they harbour the PTEN protein or not.°

**Materials and methods:**

T98G and U118MG glioblastoma cell lines were treated with the 2′3′-c-di-AM(PS)2(Rp,Rp) STING agonist together with or without the chemotherapeutic agent temozolomide. cGAS/STING pathway components were subsequently analysed using qRT-PCR, western blot, and ELISA methods.

**Results:**

Our results showed that PTEN-harbouring T98G cells responded well to STING activation, leading to increased temozolomide efficacy. In contrast, STING activation in U118MG cells did not affect the response to temozolomide. mRNA expression levels of *STING*, *IRF3*, *NF-KB*, and *RELA* genes were significantly increased at the combined treatment groups in T98G cell line. Conversely, combined treatment with STING agonist and temozolomide did not affect mRNA expression levels of cGAS/STING pathway genes in U118MG cells.

**Conclusion:**

Our data offers new evidence suggesting that STING agonists can effectively be used to increase temozolomide response in the presence of PTEN protein. Therefore, increased GBM therapy success rates can be achieved by employing the PTEN expression status as a predictive biomarker before treating patients with a chemotherapeutic agent in combination with STING agonist.

## 1. Introduction

STING is an endoplasmic reticulum resident protein that facilitates innate immunity activated by viral infections [1]. Identification of cGAS as a cytosolic DNA sensor and cGAMP production as a second messenger provided more clarification for the cGAS/STING pathway in the host immune response [2]. STING can detect genomic materials or cyclic dinucleotides (CDNs) originating from pathogens, as well as self-DNA leaked from the host nucleus or mitochondria. STING activation promptly triggers a type I interferon response immediately [3]. In addition to its crucial roles in activating innate immunity, STING-dependent cytosolic DNA sensing has also been related with immunogenicity and therapeutic sensitivity in cancer. STING activation was associated with cytotoxic T-cell infiltration and improved PARP inhibitor response [4], enhanced radiation-mediated antitumor immunity [5], or immune checkpoint blockade therapy [6]. With the discovery of CDNs as cGAS/STING pathway agonists, several companies have started to develop activator compounds to benefit from the immunomodulatory functions of STING [7].

Glioblastoma (GBM) is the most frequent and aggressive malignant primary brain tumour in adults with a progression-free survival of 14 months and 5-year overall survival of 9.8% with the current standard-of-care involving surgery followed by radiotherapy and temozolomide (TMZ), which is a DNA alkylating agent [8]. Although TMZ displays antitumor activity and limited toxicity, its survival benefit remains unsatisfactory, and over 50% of the treated patients acquire resistance to TMZ in part due to the (re)expression of a gene called *O6-methylguanine-DNA methyltransferase* [9]. Recurrence of the tumour is an inevitable event in the GBM and most patients acquire it after 6–9 months of primary treatment [10]. Phosphatase and tensin homolog (PTEN) mutations are found in 41% of GBM patients and have been linked to TMZ resistance [11–13]. The low expression of PTEN and the high expression of STING were associated with poor prognosis and shortened overall survival of patients diagnosed with tongue squamous cell carcinoma [14]. It was reported that human glioblastoma tumours express STING pathway components, i.e. STING, TBK1, and IRF-3 [15]. STING activation triggered immune surveillance and hindered tumour development through vascular disruption in in vivo GBM models [16].

GBM is characterized with immunosuppressive microenvironment; therefore, developing immunomodulatory compounds to activate the immune response is crucial for increasing success rates [17]. PTEN is one of the frequently altered tumour suppressor genes in cancers and associated with immunosuppressive tumour microenvironment [18]. During the antiviral innate immunity, PTEN controls the import of Interferon Regulated Factor 3 (IRF3) transcription factor into the nucleus to trigger interferon production [19]. Furthermore, PTEN and STING proteins are important for regulation of oxidative stress-induced liver inflammation and necroptosis in macrophage cells [20]. Therefore, we hypothesized that STING activation might generate different expression patterns and temozolomide responses in cells depending on whether they harbor the PTEN protein or not.

## 2. Materials and methods

### 2.1. Cell culture

T98G and U118MG cell lines were obtained from American Type Culture Collection (ATCC). The T98G cell line carries c.125T>G mutation in the 2nd exon of *PTEN* gene, leading to mRNA and protein overexpression [21]. In contrast, U118MG cell line carries a frame shift mutation, c.1026+1G>T, resulting in a lack of functional PTEN protein [22]. Both cell lines were grown in DMEM supplemented with 10% foetal bovine serum (FBS), L-glutamine, and penicillin-streptomycin at 37 °C under humidified atmosphere with 5% CO_2_.

### 2.2. Cytotoxicity analysis

STING agonist (SA), 2′3′-c-di-AM(PS)2(Rp,Rp), was obtained from Invivogen (#tlrl-nacda2r-01) and dissolved in water at a concentration of 50 μg/mL. Temozolomide was purchased from Sigma (#T2577) and dissolved at 50 mM concentration in DMSO. Cytotoxicity analyses were performed using xCELLigence real-time cell analyser system. T98G cells were plated at a density of 1 × 10^4^ per well, and U118MG cells were plated at a density of 7.5 × 10^3^ cells per well. After 24 h, 2 μg/mL of STING agonist or the IC_50_ dose of temozolomide was added to the wells [23]. The cell index was analysed for 72 h, and the data was evaluated using the instrument’s software.

### 2.3. qRT-PCR

All qRT-PCR primers were obtained from Oligomer Biotechnology, and SYBR Green enzyme was obtained from Bio-Rad. mRNA expression levels of *STING (TMEM173)*, *IRF3*, *NF-KB (P50)*, and *RELA (P65)* genes were analysed using quantitative qRT-PCR. Glyceraldehyde 3-phosphate dehydrogenase *(GADPH)* expression was used as the housekeeping gene for normalisation.

### 2.4. Western blot

Primary antibodies and their dilution concentrations used in the western blot analysis are as follows: beta-actin (Cell Signaling, 1/1000), STING (Cell Signaling, 1/1000), IRF3 (Cell Signaling, 1/1000), and NF-KB (Cell Signaling, 1/1000). Protein lysates were isolated using complete lysis-M buffer (Roche Applied Science), and the obtained protein amounts were assessed with the Bradford method. Subsequently, 30 μg of each protein extract was resolved in 8% SDS-PAGE gel and transferred to PVDF membranes. A western blot chromogenic detection kit (Invitrogen) was used for protein detection.

### 2.5. ELISA assay

Human interleukin (IL) 6 and IFNα (Elabscience) kits were used for ELISA analysis. Following treatment with TMZ, STING agonist, or both, cells were cultured for 48 hours, after which supernatants were collected and used for IL6 and IFNα analysis.

## 3. Results

**Figure 1:**
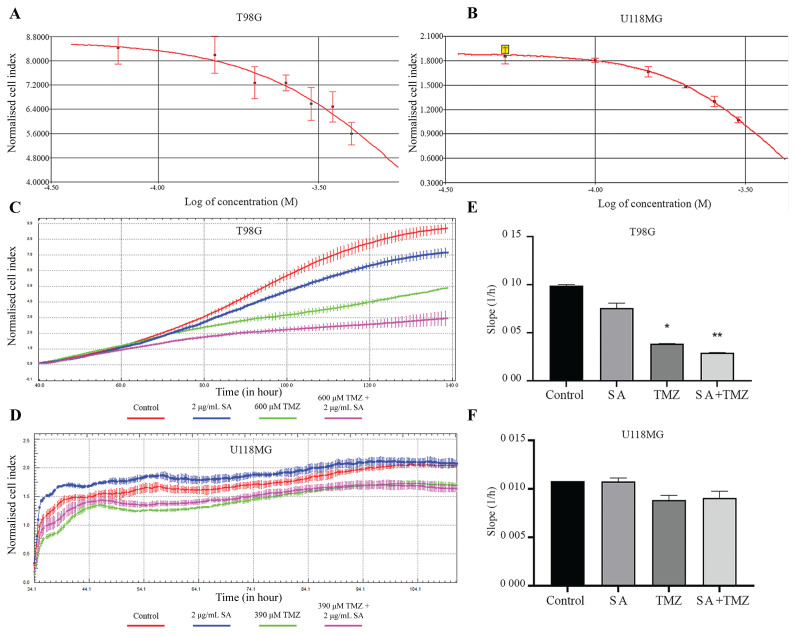
Effects of STING agonist (SA) on temozolomide (TMZ) response of T98G and U118MG cell lines. **A–B:** T98G and U118MG cells were treated with increasing concentrations of TMZ for 72 h, and IC_50_ levels were calculated using xCELLigance software. **C–D:** Cells were treated with the 2 μg/mL SA, TMZ, or both (SA + TMZ), and the cell indexes analysed over a 72-hour period. **E–F:** Slope values were obtained from xCELLigance software (*p = 0.002; **p = 0.004).

The IC_50_ concentration of temozolomide was 600 μM for the T98G cells and 400 μM for the U118MG cells (Figures 1A and 1B). To investigate the effects of the STING agonist on the response to TMZ, we treated T98G and U118MG cell lines with 2 μg/mL of SA, TMZ, and the combination of both, and analysed proliferation for the next 72 h. Combined treatment with 600 μM TMZ and 2 μg/mL SA showed more inhibitory effect on the T98G cells proliferation (Figures 1C and 1D). However, there was no significant difference between the combination therapy and temozolomide alone administration in U118MG cells (Figures 1E and 1F).

**Figure 2:**
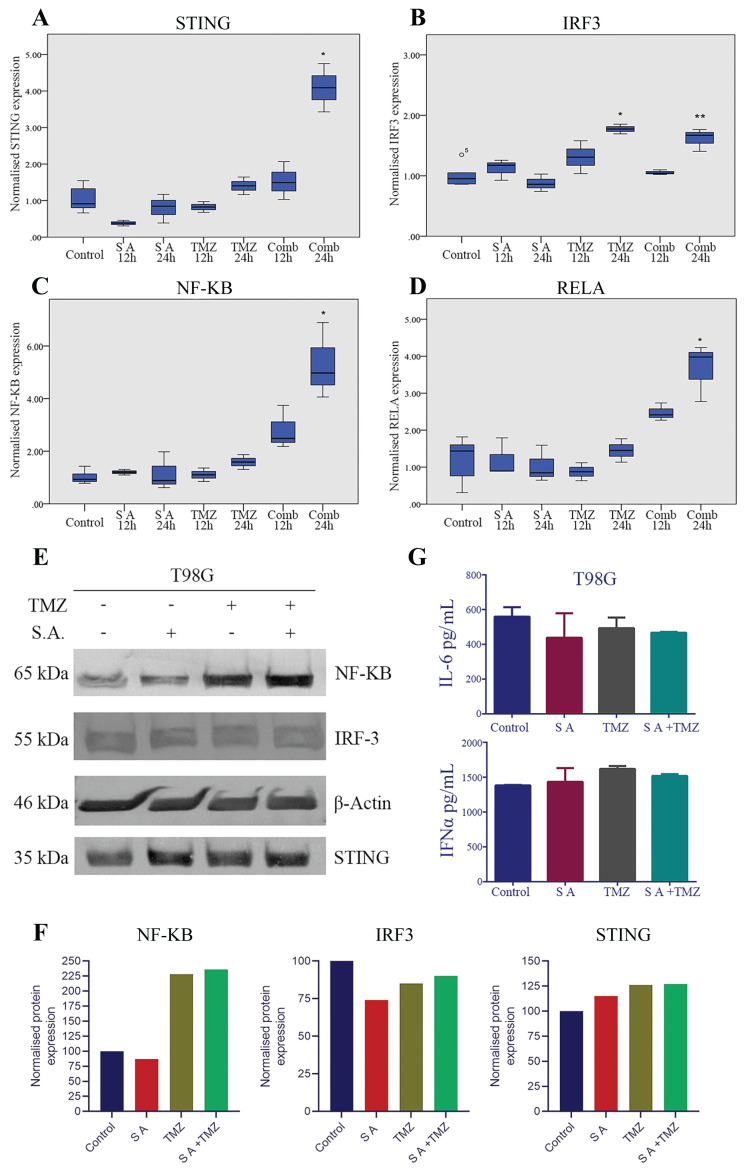
Effects of SA and TMZ treatment on cGAS/STING pathway in the T98G cell line. Normalized mRNA expressions of **A:** *STING* p = 0.045 (Control vs Comb24h), p = 0.049 (SA12h vs Comb24h), p = 0.03 (SA24h vs Comb24h), **B:** *IRF3* *p = 0.016 (SA24h vs TMZ24h) **p = 0.012 (TMZ24h vs Comb24h), **C:** *NF-KB* p = 0.001 (Control, SA12h, SA24h, TMZ12h and TMZ24h vs Comb24h), **D:** *RELA* p = 0.001 (Control, SA12h, SA24h, TMZ12h and TMZ24h vs Comb24h); **E:** Western blot results of target proteins; **F:** Relative quantification graphs of western blot results; **G:** IFNα (p = 0.088) and IL-6 (p = 0.363) expression levels.

Downstream of STING signalling, IRF3 and nuclear factor kappa B subunit 1 (NF-KB, also known as P50) transcription factors work synergistically to activate type I interferons and cytokines [24, 25]. Therefore, we analysed both to investigate whether SA and TMZ upregulates IRF3 or NF-KB-induced cytokine production. RELA (also known as P65) binds NF-KB to form the most abundant heterodimer form of NF-KB. Our results showed that *STING*, *IRF3*, *NF-KB*, and *RELA* mRNA expression levels were significantly increased at the 24 h combined treatment groups in T98G cell line (Figures 2A–2D). Western blot analysis also confirmed the elevated STING and NF-KB proteins after combined treatment (Figures 2E and 2F). When we analysed cell culture supernatants in terms of IFNα and IL-6 expression, we did not observe any significant change between treatment groups (p = 0.088 and p = 0.363; Figure 2G).

**Figure 3:**
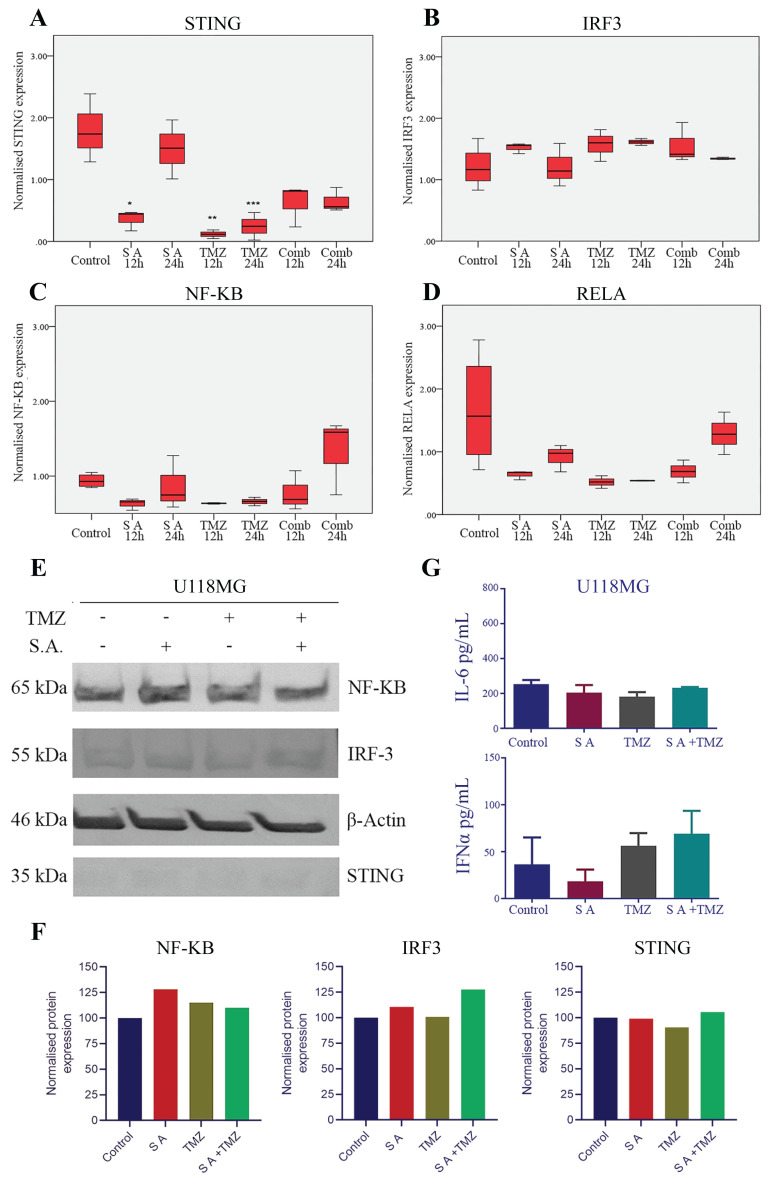
Effects of SA and TMZ treatment on cGAS/STING pathway in the U118MG cell line. Normalized mRNA expressions of **A:** *STING* *p = 0.011 (Control vs SA12), **p = 0.003 (Control vs TMZ), ***p = 0.006 (Control vs TMZ24h), **B:** *IRF3*, **C:** *NF-KB*, **D:** *RELA*, **E:** Western blot results of target proteins; **F:** Relative quantification graphs of western blot results; **G:** IFNα (p = 0.072) and IL-6 (p = 0.085) expression levels.

TMZ treatment significantly decreased *STING* mRNA expression in U118MG cells (Figure 3A). On the other hand, *IRF3*, *NF-KB*, and *RELA* expressions did not significantly change in any of the treatment groups (Figures 3B–3D). Western blot analysis showed that U118MG cells express low levels of IRF3 and STING proteins (Figures 3E and 3F). U118MG cells also showed noticeably low IFNα and IL6 levels comparing to T98G cells; however, ELISA assays did not show significant up- or downregulation between the treatment groups (p = 0.072 and p = 0.085; Figure 3G).

## 4. Discussion

In this study, we aimed to compare the effects of STING agonist 2′3′-c-di-AM(PS)2 (Rp,Rp) on PTEN-harbouring and PTEN-deficient glioblastoma cell lines in terms of response to temozolomide and cGAS/STING pathway. Several reports indicated that cGAS/STING signalling is frequently suppressed in cancers [26, 27]. Colorectal cancer patients with higher STING expression showed longer overall and recurrence-free survival; therefore, it was reported that higher STING expression may be an independent prognostic factor for overall survival [28]. STING activation was also revealed as a predictive biomarker in lung cancer to predict immunotherapy response [29].

Native and non-nucleotide agonists of STING are under development as potential agents to increase the efficacy of cancer therapy. [30]. For instance, local delivery of STING agonist with camptothecin provided tumour regression and increased animal survival [31]. IL-15 in combination with the STING agonist (ADU-S100) induced prostate cancer cell death by increasing natural killer cells [32]. Therapeutic efficacy of PARP inhibitors was associated with CD8^+^ T-cell recruitment via STING pathway activation in triple-negative breast cancer (TNBC) [4]. Similarly, the efficacy of 5-Fluorouracil was associated with antitumour immunity triggered by cancer-cell-intrinsic STING activation [33].

ASA404, also known as DMXAA, showed strong effects on subcutaneous brain tumour model but did not exhibit an activity in orthotopic model [34]. Because the signalling strength is important for proapoptotic functions of STING, low penetration of ASA404 into the brain may be responsible for insufficient effects in the intracranial tumours [35]. Boudreau et al. investigated the intratumoral administration of STING agonist (IACS-8779) to canine glioblastoma and reported well toleration up to 15 μg and higher doses were associated with radiographic responses [36]. Immunostimulatory mesoporous silica nanoparticles (immuno-MSN) carrying cyclic diguanylate monophosphate (cdGMP) and STING agonist were systemically delivered and facilitated circulating CD8^+^ T-cell activity and delayed tumour growth in a mouse GBM model [37]. Combination therapy of anti-CD47 antibodies and STING agonists increased the macrophage polarization to M1-phenotype, reduced tumour immunosuppression, and inhibited the orthotopic GBM growth [38]. These results from glioblastoma models indicate a potential use of STING agonists in enhancing the efficacy of immunotherapy and other treatments by shifting the tumour microenvironment towards to the immune active phenotype. In this study, we combined STING agonist with temozolomide, and compared the treatment response according to the PTEN genotype. Our results showed that PTEN-expressing cells better responded to the combination treatment of STING agonist and temozolomide, whereas STING agonist did not change the temozolomide response of PTEN-deficient cells.

PTEN is a dual phosphatase that has key functions in several cell regulatory mechanisms and tumour suppression. It was reported that PTEN controls the import of the IRF3 transcription factor, which is responsible for the IFN response, into the nucleus [19]. PTEN-deficient cancers are associated with an immunosuppressive tumour microenvironment [18]. Molecular determinants of immunotherapeutic response in GBM were reported as specific molecular alterations, immune expression signatures, and immune infiltration that reflect the tumour’s clonal evolution during treatment [39]. Different therapy strategies for GBM tried so far have failed to improve survival in randomized clinical trials, and the standard of care has remained unchanged over the last decade [9]. Therefore, STING agonists have significant potential for the development of GBM therapy and hold promise for the invention of new treatment combinations in the near future.
